# *Penicillium excelsum sp*. *nov* from the Brazil Nut Tree Ecosystem in the Amazon Basin’

**DOI:** 10.1371/journal.pone.0143189

**Published:** 2015-12-30

**Authors:** Marta Hiromi Taniwaki, John I. Pitt, Beatriz T. Iamanaka, Fernanda P. Massi, Maria Helena P. Fungaro, Jens C. Frisvad

**Affiliations:** 1 Centro de Ciência e Qualidade de Alimentos, Instituto de Tecnologia de Alimentos, Campinas, São Paulo, Brazil; 2 CSIRO Food and Nutrition, North Ryde, New South Wales, Australia; 3 Centro de Ciências Biológicas, Universidade Estadual de Londrina, Londrina, Paraná, Brazil; 4 Department of Systems Biology, Technical University of Denmark, Lyngby, Denmark; University of Sydney, AUSTRALIA

## Abstract

A new *Penicillium* species, *P*. *excelsum*, is described here using morphological characters, extrolite and partial sequence data from the ITS, β-tubulin and calmodulin genes. It was isolated repeatedly using samples of nut shells and flowers from the brazil nut tree, *Bertolletia excelsa*, as well as bees and ants from the tree ecosystem in the Amazon rainforest. The species produces andrastin A, curvulic acid, penicillic acid and xanthoepocin, and has unique partial β-tubulin and calmodulin gene sequences. The holotype of *P*. *excelsum* is CCT 7772, while ITAL 7572 and IBT 31516 are cultures derived from the holotype.

## Introduction

*Penicillium* species are very important agents in the natural processes of recycling biological matter. Some species cause deterioration of all sorts of man-made goods; some rot fruit or spoil foods; some species secrete secondary metabolites (extrolites) such as mycotoxins (*e*.*g*. ochratoxins, patulin, citrinin), while other extrolites are used as pharmaceuticals, including antibiotics such as penicillin and the cholesterol-lowering agent lovastatin [1, 2, 3, 4]. Some species are known for their production of organic acids and diverse enzymes that degrade a wide variety of complex biomolecules [1, 2, 3]. A variety of species are capable of producing or modifying biological chemicals, and this field is set for great expansion. A few species are directly involved in food production: this field is not likely to expand, because many species produce mycotoxins. *Penicillium* is an ascomycete genus and belongs to the family *Aspergillaceae* [4]. More than 350 species are currently accepted in this genus [5].

The Amazon rainforest has multiple ecosystems with a huge fungal biodiversity. It has an important role in the global weather balance and is the location of many native people. The equatorial climate is hot and humid, with an average temperature of 26°C and relative humidity 80–95%.

Brazil nuts are one of the most important products taken from the Amazon rainforest region. Brazil nut trees, *Bertholletia excelsa* Humb. & Bonp., grow wild, take 12 years to bear fruit, may live up to 500 years and reach up to 60 m high. Pollination of the unusual flowers requires wild, large bodied bees, especially from the family *Euglossinae* [6]. The fungal species most commonly isolated from brazil nuts are *Aspergillus flavus*, *A*. *nomius*, *A*. *pseudonomius*, *A*. *niger*, *A*. *tamarii*, *Penicillium glabrum*, *P*. *citrinum*, *Rhizopus* spp., *Fusarium oxysporum* [7, 8, 9, 10, 11, 12] and *A*. *bertholletius*, a species described recently [13].

During a study of the mycobiota of the brazil nut tree ecosystem, including flowers, brazil nuts, soil, bees and ants, an undescribed *Penicillium* species was found. This species is described here as *Penicillium excelsum sp*. *nov*.

## Materials and Methods

### Sample collection, isolation and morphological examination

Samples were collected from the ecosystem of the brazil nut tree, *Bertholletia excelsa* in the Amazon rainforest in Para and Amazon States, Brazil. Sample collection and methodology have been described previously [13]. Briefly, samples of brazil nut kernels and shells, flowers and leaves, soil from beneath the trees, plus bees and ants. Collecting was carried out in collaboration with the Brazilian Ministry of Agriculture.

For fungal isolation, nuts and shell samples were disinfected in sodium hypochlorite solution, then plated onto dichloran 18% glycerol agar (DG18), according to the methodology of Pitt and Hocking [2]. Soil samples were mixed with sterile water containing peptone (0.1%), then serially diluted and spread plated onto DG18. Flower and leaf samples were surface disinfected as above and plated onto DG18 while bee and ant samples were plated on DG18 without surface disinfection. All plates were incubated at 25°C for 7 days, then all colonies of *Penicillium* species were transferred onto Czapek yeast extract agar [2] and incubated at 25°C for 7 days for further identification.

The *Penicillium* isolates were examined on standard identification media for *Penicillium* species according to Pitt [14] namely: Czapek yeast extract agar (CYA), malt extract agar (MEA, Oxoid), and 25% glycerol nitrate agar (G25N) at 25°C and also on CYA at 37°C and 42°C, plus oatmeal agar (OAT), creatine sucrose agar (CREA) and yeast extract sucrose (YES) agar [3]. The incubation time for all media was 7 days and plates incubated in the dark.

The standard conditions used for the description of *Penicillium excelsum* are taken from Pitt [14] and Frisvad and Samson [15]. Capitalized colours are from the Methuen Handbook of Colour [16].

### DNA extraction, amplification, sequencing and phylogenetic analysis

A standard phenol:chloroform extraction protocol [17] was used for genomic DNA isolation from an extype culture (ITAL 7572). The primer-pairs ITS1-ITS4 [18], Bt2a-Bt2b [19] and cmd5-cmd6 [20] were used to amplify the ITS1-5,8S-ITS2 region (ITS), partial β-tubulin gene (*BenA*) and partial calmodulin gene (*CaM*) respectively, adopting a standard amplification cycle, which ran 35 cycles with an annealing temperature of 55°C [5]. Excess primers and dNTPs were removed from the PCR product using the Wizard® SV Gel and PCR Clean-Up System (Promega, Wisconsin, USA). Purified PCR products were sequenced in both directions using a BigDye® Terminator v3.1 Cycle Sequencing kit (Applied Biosystems, California, USA) according to the manufacturer’s instructions. A volume of HiDiformamide (10 μl) was added to the sequencing products, which were processed in an ABI 3500XL Genetic Analyzer (Applied Biosystems). Contigs were assembled using the forward and reverse sequences with the programme SeqMan from the Laser Gene package (DNAStar Inc., Wisconsin, USA). All sequences were subjected to Basic Local Alignment Search Tool (BLAST) against the NCBI database to identify *Penicillium* species with similar DNA sequences. The ITS and *BenA* sequences were aligned by ClustalW algorithm using Mega5.1 software (21) with those from *Penicillium* subgenus *Aspergilloides* section *Lanata*-*Divaricata* type or neotype strains, as recently suggested by Visagie et al [5]. Phylogenetic trees were constructed with Mega5.1 software [21], using the Neighbor-Joining (NJ) and Maximum Likelihood (ML) methods based on the Tamura-Nei model [22]. To determine the support for each clade, a nonparametric *bootstrap* analysis was performed with 1,000 resamplings.

The ITS, *BenA* and *Ca*M sequences were deposited in GeneBank under the respective following accession numbers: KR815341, KP691061, KR815342 (strain ITAL 7572); KT749964, KT749957, KT749961 (strain ITAL 7814); KT749965, KT749958, KT749960 (strain 7823); KT749963, KT749959, KT749962 (strain 7804).

### Extrolite analysis

Cultures were analysed by High Performance Liquid Chromatography (HPLC) with a diode array detector (HPLC-DAD) as described by Frisvad and Thrane [23] and modified by Houbraken et al. [24], as previously described [13]. Three agar plugs each from CYA and YES medium were pooled and extracted with 0.75 mL of a mixture of ethyl acetate/ dichloromethane/methanol (3:2:1) (v/v/v) with 1% (v/v) formic acid.

### Nomenclature

1. The new name contained in this work has been submitted to MycoBank from where it will be made available to the Global Names Index. The unique MycoBank number can be accessed and the associated information viewed through any standard web browser by appending the MycoBank number contained in this publication to the prefix http://www.mycobank.org/MB811066.
2. Repository of ***Penicillium excelsum*** Taniwaki, Pitt & Frisvad 2015 sp. nov. [urn:lsid:mycobank.org: 811066]

## Results and Discussion

### Sources of the isolates

In total, 116 isolates of -the new species described here as *Penicillium excelsum* were found in brazil nut shells and kernels, from soil close to *Bertholletia excelsa* trees, and from flowers, bees and ants associated with *Bertholletia* trees. The origins of representative *P*. *excelsum* isolates are shown in Table 1. Soil may be the primary habitat of this species, as many species of *Penicillium* are soil fungi [4, 25]. However, this study shows that *P*. *excelsum* also occurs on bees and ants, which may carry spores to the flowers, and other locations by contact or excreta which will all play a role in dispersal of this species.

**Table 1:** *Penicillium excelsum* isolates from the Amazon region.

N°	Substrate	Rainforest	Sample n°
ITAL 2172	Flowers	Amazon	85
ITAL 2248	Flowers	Amazon	96
ITAL 3000/IBT 30867	Nut	Pará	113
ITAL 3030/IBT 30865	Flowers	Pará	132
ITAL 3743	Nut	Pará	120
ITAL 3904	Flowers	Pará	136
ITAL 3931	Flowers	Pará	137
ITAL 3985	Flowers	Pará	142
ITAL 4005	Flowers	Pará	143
ITAL 4067	Flowers	Pará	144
ITAL 4419	Nut	Pará	152
ITAL 4432	Nut	Pará	153
ITAL 4451	Flowers	Pará	155
ITAL 4493	Nut	Pará	164
ITAL 4545	Flowers	Pará	165
ITAL 4570	Flowers	Pará	166
ITAL 6705	Nut	Amazon	211
ITAL 6706	Shell	Amazon	211
ITAL 6722	Nut	Amazon	212
ITAL 6752	Nut	Amazon	213
ITAL 7014	Nut	Amazon	220
ITAL 7035	Shell	Amazon	220
ITAL 7572*/CCT 7772/IBT 31516	Shell	Amazon	234
ITAL 7613	Shell	Amazon	236
ITAL 7732	Flowers	Amazon	239
ITAL 7741/CCT 7773/IBT 32953	Flowers	Amazon	240
ITAL 7760/CCT 7775/IBT 32732	Flowers	Amazon	240
ITAL 7770/CCT 7776	Flowers	Amazon	241
ITAL 7788/CCT 7777	Flowers	Amazon	242
ITAL 7804/CCT 7778	Flowers	Amazon	242
ITAL 7814/CCT 7779	Bees	Amazon	244
ITAL 7823/CCT 7780	Bees	Amazon	244
ITAL 7875/CCT 7781	Ants	Amazon	250
ITAL 8014	Soil	Amazon	255

* Type culture

Culture collection of: Instituto de Tecnologia de Alimentos (ITAL), Coleção de Cultura Tropical (CCT), Technical University of Denmark (IBT).

### Extrolites

**Fig 1:**
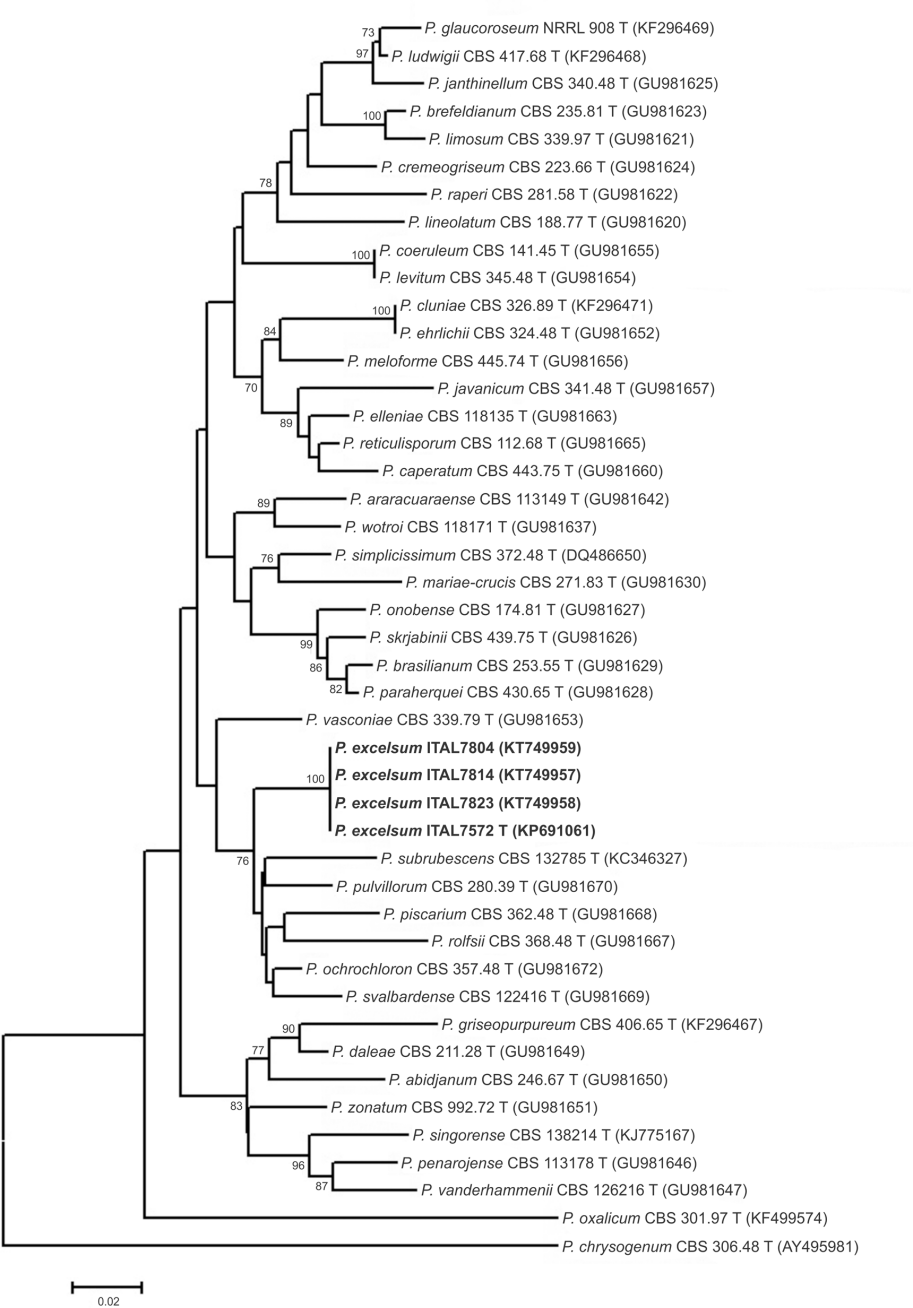
Neighbour joining tree reconstructed from the partial β-tubulin (*BenA*) gene sequences aligned with corresponding sequences of *Penicillium* section *Lanata-Divaricata* deposited in public databases. Numbers at branch nodes refer to bootstrap values (1000 replicates), only values of >70% are shown.

**Fig 2:**
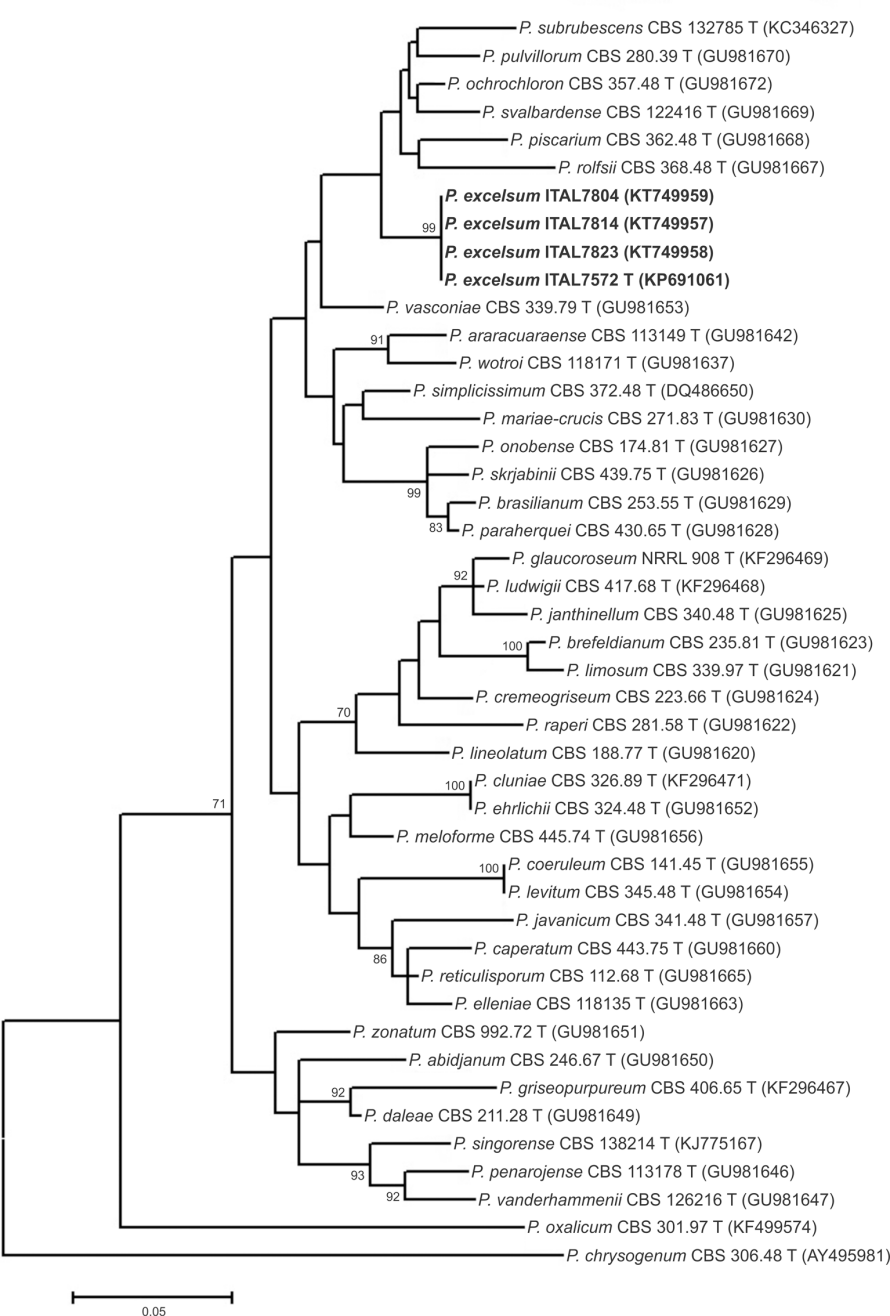
Maximum-likelihood tree reconstructed from the partial β-tubulin (*BenA*) gene sequences aligned with corresponding sequences of *Penicillium* section *Lanata-Divaricata* deposited in public databases. Numbers at branch nodes refer to bootstrap values (1,000 replicates), only values of >70% are shown.

**Fig 3:**
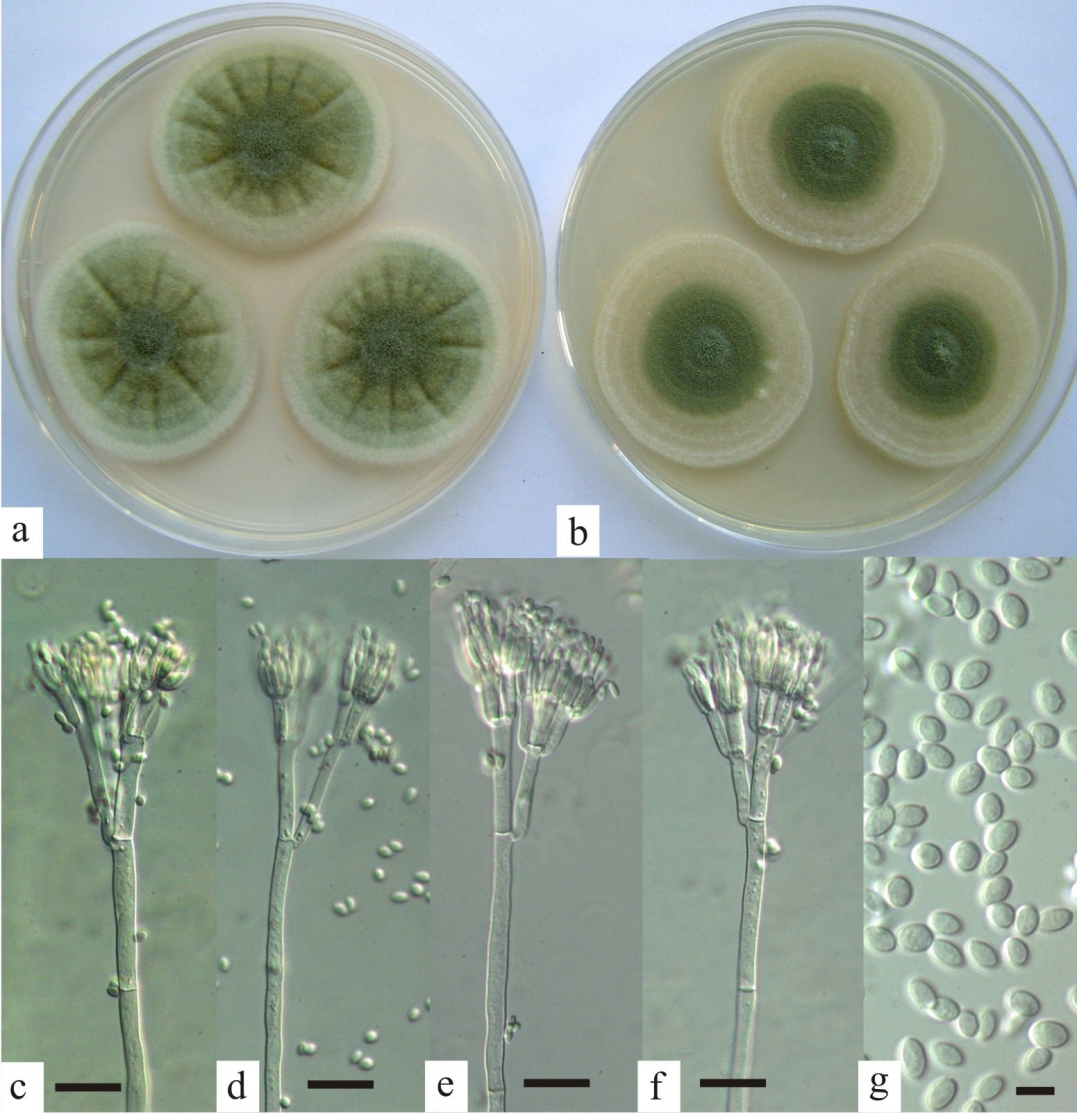
Penicillium excelsum. Colonies after 7 days at 25°C on (a) Czapek yeast extract agar; (b) malt extract agar; (c–f) penicilli, bar = 20 μm; (g) conidia, bar = 5 μm.

HPLC-DAD analysis of extracts showed that several strains of *P*. *excelsum* produce andrastin A, penicillic acid, while some also produce xanthoepocin. Strain ITAL 3000 also produced curvulic acid. Related species also produce penicillic acid, for example *P*. *brasilianum*, *P*. *cremeogriseum*, *P*. *ochrochloron P*. *pulvillorum* and *P*. *vanderhammenii* [24, 26, 27]. *P*. *pulvillorum* and *P*. *simplicissimum* have also been reported to produce andrastin A, and *P*. *brasilianum*, *P*. *ochrochloron*, *P*. *pulvillorum*, *P*. *rolfsii*, *P*. *simplicissimum* and *P*. *svalbardense* have been reported to produce xanthoepocin [24, 28]. Even though andrastin A, penicilllic acid, and xanthoepocin have been found in species outside section *Lanata-Divaricata* [15] the particular combination of these extrolites is mostly found in this section. *P*. *excelsum* produces a profile of extrolites close to that of *P*. *brasilianum*, *P*. *ochrochloron*, *P*. *pulvillorum* and *P*. *rolfsii* and the close relationship is confirmed by sequence and morphological data as shown in Figs 1, 2 and 3.

### Phylogenetic analyses

*P*. *excelsum* ITS, *BenA* and *CaM* sequences were found to be different from all other sequences in NCBI (accessed 30 May, 2015). When the BLAST searches were performed using the option “sequences from type material” [29] the sequences harmonized in showing that *P*. *excelsum* is most similar to *P*. *ochrochloron* neotype strain CBS 357.48 and *P*. *pulvillorum* neotype CBS 280.39. Both, *P*. *pulvillorum* and *P*. *ochrochloron* belong to *Penicillium* subgenus *Aspergilloides* section *Lanata-Divaricata* in the recent phylogenetic reclassification of *Penicillium* [4].

A more recent study [5] provided GenBank accession numbers to reference sequences for all accepted *Penicillium* species. Using these reference sequences, ITS-based phylograms (data not shown) generated using Neighbor-Joining and Maximum Likelihood techniques confirmed the placement of *P*. *excelsum* in section *Lanata-Divaricata*. Although the ITS phylograms of *P*. *excelsum* clustered and were differentiated from other species of section *Lanata-Divaricata*, the majority of bootstrap values of branches were low, meaning that the ITS tree was poorly resolved. The ITS region is accepted as the primary fungal barcode [30]; however, it is well known that the ITS region provides only poor resolution of many *Penicillium* species [5, 31]. In consequence, it has been proposed [5] that β-tubulin (*BenA*) is an optimal secondary identification marker for *Penicillium* species. *BenA*-based phylograms, generated using both Neighbor-Joining and Maximum Likelihood methods, placed *P*. *excelsum* on a branch separated from all other species on *Penicillium* section *Lanata-Divaricata* (Figs 1 and 2). Neighbor-Joining and Maximum Likelihood based phylograms were consistent and reveled that *P*. *excelsum* represent a separated lineage within a clade composed of *P*. *pulvillorum*, *P*. *svalbardense*, *P*. *piscarium*, *P*. *ochrochloron*, *P*. *rolfsii*, and *P*. *subrubescens*.

### Taxonomy

***Penicillium excelsum* sp. nov.** Taniwaki, Pitt & Frisvad sp. nov. Mycobank MB 811066 (Fig 3)

On CYA at 7 days, 25°C, colonies 35–50 mm in diameter, dense, lightly sulcate, mycelium white to off-white; lightly sporing, coloured greenish grey (M. 28D2); exudate and soluble pigment absent; reverse pale to slightly brown or pink.

On MEA at 7 days, 25°C, colonies 28–50 mm in diameter, low and sparse, plane, mycelium hyaline to white; lightly sporing, greenish grey; exudate and soluble pigment absent; reverse pale brown.

On G25N at 7 days, 25°C, colonies 10–14 mm in diameter, low and dense, coloured buff with light sporulation; reverse brown to deep brown.

On YES agar at 7 days, 25°C, colonies 34–42 mm in diameter, moderate sporulation and a brown reverse.

On OAT at 7 days, 25°C, colonies 41–48 mm, strong sporulation, reverse brown.

On CREA at 7 days, 25°C, colonies 18–32 mm, weak growth, no sporulation, and no acid production.

At 37°C on CYA, colonies 8–22 mm in diameter, coloured grey to brown; soluble pigment brown, reverse deep brown.

At 42°C on CYA, no growth.

Conidiophores borne from surface hyphae, long and robust, up to 600 x 4.0–5.0 μm, smooth walled, septate, bearing typically biverticillate appressed penicilli, metulae commonly 15–18 x 4–6 μm, but sometimes terverticillate with rami 15–40 μm long; phialides appressed, 10–12 x 3.0–3.5 μm, ampulliform-acerose, bearing ellipsoidal conidia, 4.0–5.0 x 2.0–3.2 μm, smooth walled.

### Holotype

CCT 7772, a freeze dried culture in Coleção de Cultura Tropical (Campinas, Brazil) is designated as the holotype of *P*. *excelsum*. It was isolated from brazil nut shell, Amazon, Brazil, 2011, by Taniwaki, M.H.

Cultures derived from this type include ITAL 7572 (where ITAL stands for the culture collection of Instituto de Tecnologia de Alimentos, Campinas, Brazil, accredited as Faithful Depositary by the Brazilian Executive Secretary of the Board of the Genetic Heritage Management n° 125/2015) and IBT 31516 (where IBT is the culture collection of the Technical University of Denmark, Lyngby, Denmark).

Etymology: named for the Amazonian brazil nut tree, *Bertholletius excelsa*, with which this species is associated.

Other isolates examined. ITAL 3000 (= IBT 30867), ITAL 3030 (= IBT 30865), ITAL 7741 (= CCT 7773 = IBT 32953), ITAL 7760 (= CCT 7775 = IBT 32732), ITAL 7770 (= CCT 7776), ITAL 7788 (= CCT 7777), ITAL 7804 (= CCT 7778), ITAL 7814 (= CCT 7779), ITAL 7823 (= CCT 7780) and ITAL 7875 (= CCT 7781).

### Distinguishing features

This species is classified in *Penicillium* subgenus *Furcatum* section *Furcatum* in the classification of Pitt [14] and *Penicillium* subgenus *Aspergilloides* section *Lanata-Divaricata* according to Houbraken and Samson [4].

Morphologically, *P*. *excelsum* differs from the closely related *P*. *subrubescens*, *P*. *pulvillorum*, *P*. *piscarium*, *P*. *rolfsii*, *P*. *ochrochloron* and *P*. *svalbardense* by having a combination of smooth stipes, the frequent formation of rami, and the production of large, ellipsoidal, smooth walled conidia. *P*. *ochrochloron* and *P*. *rolfsii* are similar, but have finely roughened conidia. *P*. *subrubescens*, *P*. *pulvillorum*, *P*. *piscarium* and *P*. *svalbardense* produce globose to subglobose conidia, and in addition the conidia of *P*. *piscarium* are distinctly rough-walled. *P*. *excelsum* grows well at 37°C, though not as well as *P*. *rolfsii*. Most isolates of *P*. *subrubescens* and *P*. *pulvillorum* produce a red reverse colour on malt extract agar, whereas the reverse of *P*. *excelsum* is pale brown.

**Fig 4:**
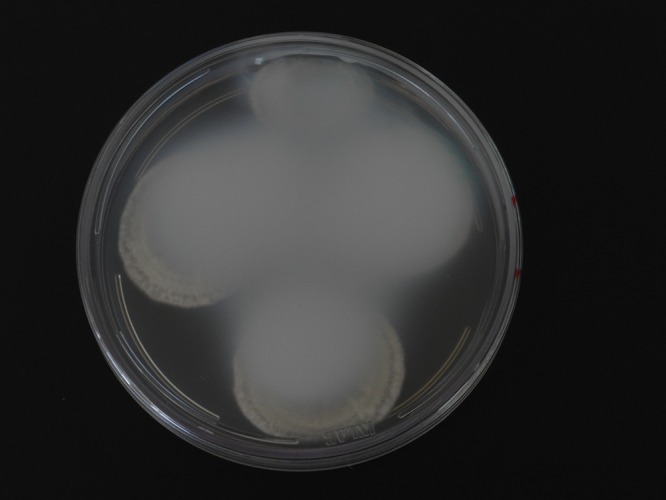
Plate of Czapek yeast extract agar (CYA) with *Penicillium excelsum*, opaqueness of petri dish lid after 7 days of incubation at 25°C.

This species is also distinguished by a unique profile of extrolytes and by unique DNA sequences in the ITS, *BenA* and *CaM* genes. This species is also notable in that cultures on CYA, MEA and YES agar cause the polystyrene plastic in Petri dishes to become opaque over time (Fig 4). The opaqueness cannot be removed using a scapel, as the chemical reaction with the plastic lids was irreversible. A volatile compound produced by the fungus as it grows must be responsible. A preliminary examination of the volatiles from *P*. *excelsum* showed that it produced large amounts of acetic acid. An HPLD-DAD analysis of the opaque layer on the Petri dish lid revealed no detectable extrolites, indicating that the compound responsible for the opaqueness is without a chromophore. This effect has not been reported from any *Penicillium* or *Aspergillus* species. Further studies will be carried out in order to determine these compounds.

## Conclusion

*P*. *excelsum* represents a new important phylogenetic species after applying a polyphasic approach using morphological characters, extrolite data, ITS, *BenA* and *CaM* partial sequences. *P*. *excelsum* is distinguished by a combination of a unique profile of extrolites, DNA sequence, micro-morphological features and the unique capacity to render Petri dish lids irreversible opaque.
